# A Cross-Sectional Investigation of the Quality of Selected Medicines for Noncommunicable Diseases in Private Community Drug Outlets in Cambodia during 2011–2013

**DOI:** 10.4269/ajtmh.19-0247

**Published:** 2019-09-16

**Authors:** Mohammad Sofiqur Rahman, Naoko Yoshida, Hirohito Tsuboi, Uy Sokchamroeun, Tep Keila, Tey Sovannarith, Heng Bun Kiet, Eav Dararath, Yoshio Akimoto, Tsuyoshi Tanimoto, Kazuko Kimura

**Affiliations:** 1 Medi-Quality Security Institute, Kanazawa University, Kanazawa, Japan;; 2 Department of Pharmacy, University of Asia Pacific, Dhaka, Bangladesh;; 3 Department of Clinical Pharmacy and Healthcare Sciences, Kanazawa University, Kanazawa, Japan;; 4 National Health Product Quality Control Center, Ministry of Health, Phnom Penh, Cambodia;; 5 Department of Drugs and Food, Ministry of Health, Phnom Penh, Cambodia;; 6 Pharmaceutical and Medical Device Regulatory Science Society of Japan, Osaka, Japan

## Abstract

Although the issue of substandard and falsified medicines is quite well known, most research has focused on medicines used to treat communicable diseases, and relatively little research has been carried out on the quality of medicines for noncommunicable diseases (NCDs). This study was designed to assess the quality of seven widely used medicines for NCDs in Cambodia during 2011–2013. Medicines were collected from private community drug outlets in Phnom Penh (urban area), by stratified random sampling and in Battambang, Kandal, Kampong Speu, and Takeo (rural areas) by convenience sampling. Samples were subsequently analyzed by visual inspection, authenticity investigation, and pharmacopoeial analysis by high-performance liquid chromatography. Various discrepancies were observed in visual inspection of packages and medicines. Of 372 tablet/capsule samples from 64 manufacturers in 16 countries, the manufacturers confirmed 107 (28.8%) as authentic; the authenticity of other samples could not be verified. Three hundred sixty-four (97.8%) samples were registered in Cambodia. Among all samples, 23.4% (95% CI 19.2–28.0) were noncompliant in one or more of the quality tests: 12.9% (95% CI 9.7–16.7) contained an amount of active pharmaceutical ingredient outside the permitted range, including some showing extreme deviations, 14% (95% CI 10.6–17.9) failed because of content variation, and 10.8% (95% CI 7.8–14.4) failed to meet pharmacopoeial reference ranges in dissolution tests. Pharmaceutical quality appeared to be unrelated to storage conditions. Although no sample was obviously falsified, there is a high prevalence of substandard medicines for NCDs in Cambodia, indicating the need for focused regulatory action, including collaborative initiatives with manufacturers.

## INTRODUCTION

Access to quality medicines is fundamental to the effort to reduce global morbidity and mortality, and therefore, it is extremely important to prevent the distribution and sale of substandard and falsified medicines (SFs).^1–3^ In addition to economic losses, poor-quality medicines may prolong patients’ illness and even lead to death.^4,5^ In early studies of poor-quality medicines, there were disputes about definitional issues and intellectual property rights.^6^ However, in 2017, the WHO adopted the term “SFs,” replacing the ambiguous term “counterfeit medicines” as well as the provisional term “substandard/spurious/falsely labelled/falsified/counterfeit medical products.”^7^ The current WHO definitions are as follows: substandard medicines are authorized medical products that fail to meet either their quality standards or specifications, or both, whereas falsified medicines are those that deliberately/fraudulently misrepresent their identity, content, or source.^8,9^

Access to medicines in developing countries has improved in recent decades,^10,11^ although quality testing of selected pharmaceuticals in Southeast Asia has indicated that more inspection and monitoring of medicines in the region is necessary.^12–14^ A recent study by the WHO estimated that in low- and middle-income countries (LMICs), 10.5% of the medicines are substandard or falsified.^1^ In particular, there is little information about the quality of medicines for noncommunicable diseases (NCDs), although sporadic reports suggest that there are significant problems.^14–18^ In this context, the global action plan of the WHO calls for a 25% reduction in premature mortality from NCDs by 2025, whereas the United Nations Sustainable Development Goals call for a reduction of one-third by 2030.^19,20^ According to WHO estimates, 85% of worldwide deaths from NCDs occur in LMICs.^18,21,22^ Thus, to achieve universal access to safe, effective, quality, and affordable essential medicines, it will be necessary to focus on safeguarding medicines for both NCDs and communicable diseases.^23,24^

Recent studies of some established medicines have shown that the incidence of substandard products in Cambodia has declined in recent years, which can be attributed to the strong action of the government of Cambodia, with international cooperation.^25^ However, substandard and falsified medicines remain on sale in Cambodia,^26,27^ and the status of antimicrobials as well as other essential medicines for communicable and NCDs remains to be fully investigated.^27,28^ Therefore, the aim of this project was to investigate the status of selected medicines for NCDs in the private market in Cambodia, to provide data for guiding further actions and countermeasures to reduce the prevalence of SFs among these medicines.

## MATERIALS AND METHODS

### Approval.

This project was a collaborative effort between the Ministry of Health (MoH)/Department of Drugs and Food (DDF) Cambodia, the WHO-Western Pacific Region, and the Department of Drug Management and Policy, Kanazawa University, Institute of Medical, Pharmaceutical and Health Sciences, Kakuma-machi, Kanazawa city, Japan 920-1192, represented by Professor Dr. K. K. Ethical approval was not needed for this study in Cambodia, although more recently ethical review for such studies has been suggested in the literature.^29^ Regulatory approval was obtained from the respective institutions and final reports for each year have been submitted to both the WHO and MoH, Cambodia.

### Study design, area, and sample collection.

The design and analytical methods used in this study followed as far as possible the reported guidelines, including Strengthening the Reporting of Observational Studies in Epidemiology (STROBE), Medicine Quality Assessment Reporting Guidelines (MEDQUARG), and a recently published checklist of criteria for designing and reporting of medicine quality studies.^30–33^ However, as target medicines were sometimes not available, or were only available in insufficient quantity, in the listed shops, and we could not obtain a complete list of outlets, it was not feasible to follow a preplanned sampling protocol.

Samples were collected in Phnom Penh, the capital of Cambodia (urban area) and in Battambang, Kandal, Kampong Speu, and Takeo (rural areas). A list of outlets was available only for Phnom Penh (provided by DDF, MoH of Cambodia), and a stratified random sampling scheme based on random number tables was used in this area. In rural areas, we used a convenience sampling strategy, and samples were collected from outlets selected by the sampling team using a mystery shopper approach where the sampler acted as a customer.^34^ In urban areas, a drug inspector accompanied the samplers. All outlets (Pharmacy, run by a registered pharmacist; Dépôt A, run by an assistant pharmacist with 3 years’ pharmacy training; and Dépôt B, run by a retired midwife or nurse) were eligible for inclusion in the study. The samples were purchased based on the availability of the medicine of choice in the shop (Supplemental File 7), and for some samples, higher number of samples were purchased to acquire the target number of samples. A few samples were also collected from wholesalers.

Cimetidine, sildenafil, amlodipine, esomeprazole, rabeprazole, glibenclamide, and metformin were selected as candidate medicines as suggested by the DDF, Cambodia. Among these, all medicines are included in the list of essential medicines in Cambodia, and amlodipine, glibenclamide, and metformin are included in the WHO list of essential medicines. The samples were collected in June 2011, June 2012, and August 2013 by two teams, each containing one or two Japanese researcher(s), one local assistant, and one supervisor from DDF. The locally recruited members were provided with training before sampling and instructed how to purchase medicines. The storage conditions (temperature and humidity) of samples were measured with a digital thermometer and a hygrometer by another sampler during the purchase of samples. Medicines collected from the same outlet and labeled with the same international nonproprietary name, brand name, strength, size, batch/lot number, and manufacturing and expiry dates were considered as one sample. The outlet type, date of purchase, price paid, brand name, formulation, batch number, date of manufacture, and expiry date were recorded using a standard sampling form for every sample purchased. Every sample was placed in an individual ziplock bag together with the recoded data and securely stored in an air-conditioned room (20–25°C) until analysis.

### Analysis.

Analysis of the samples was carried out each year immediately after collection of the samples. Sample analysis consisted of observation of the packaging and strips, authenticity investigation of the product by the manufacturer, legality investigation of the manufacturers by medicine regulatory authorities (MRAs), registration verification of the product in Cambodia, and pharmacopoeial analysis. Details of the packaging condition and label information were noted carefully. During observation, we examined the packaging and labeling, physical appearance of the tablet/capsule, their size, shape, color, etc. according to the WHO guideline and the International Pharmaceutical Federation (FIP) checklist for visual inspection of medicines.^35,36^

### Authenticity, legitimacy investigation, and registration verification.

For the authenticity investigation, a detailed questionnaire was sent to each manufacturer and the manufacturing country to confirm the authenticity of the product and the legitimacy of the manufacturer. Each questionnaire provided detailed information about the product, including manufacturer, batch number, date of manufacture and expiry date, dosage, and strength of the product, as recommended by the WHO.^35^ The registration status of each product was evaluated by visual inspection of the packaging and then sending a questionnaire to the importing country to confirm the registration of the product.^37^ Samples were also checked for compliance with the Association of Southeast Asian Nations Common Technical Dossier for the registration of pharmaceuticals for human use (to which drug registration in Cambodia conforms).^38^

### Pharmaceutical analysis.

Pharmaceutical analysis of the samples was performed according to the pharmacopoeia specified in the sample package of the respective dosage form for each of the seven medicines. The pharmacopoeial quality assessment included identification, assay, content uniformity test, and dissolution test. Following the British Pharmacopoeia (BP) and the United States Pharmacopeia (USP) (all of the samples were labeled as BP or USP), active ingredients of the samples were quantified by high-performance liquid chromatography using ultraviolet and photo-diode array detectors (Shimadzu, Kyoto, Japan).^39–41^ The chromatographic conditions for assay and dissolution tests are provided in Supplemental Table 1.

Mechanical calibration and performance verification test were performed before sample testing for performance qualification and to ensure the absence of technical and mechanical errors. Test methods and system suitability for each medicine were validated according to USP 34.^39^ A linear relationship between the peak area and concentration of each reference standard was established within the range of 25–200% of the active ingredient (*r*^2^ = 0.999–1.000), and the assay was performed within that range. The intra- and inter-day coefficients of variation were less than 3.0%. In addition, the methods were validated for accuracy and precision (*n* = 6). The samples were analyzed in Kanazawa University after collection in each year. All the quality analyses were completed before the expiry date of the samples. Identification, assay, content uniformity, and dissolution test were performed according to USP 34, USP 35, or BP 2012 for all the samples as indicated on the package insert or outer package, except in the cases of sildenafil and rabeprazole.^39–41^ The analytical method for sildenafil was developed according to the method suggested by Moriyasu et al.^42^ The method for rabeprazole was developed based on the approval information document of rabeprazole provided by the Pharmaceuticals and Medical Devices Agency (PMDA).

### Definition of compliance of samples with standards.

Samples were evaluated as meeting the quality specifications if the amount of active pharmaceutical ingredient (API) in each of the units, as determined from their content uniformity test, lay within the range of 90.0–110.0% of the label claim. For content uniformity, acceptance value was calculated according to USP 34.^39^

In the dissolution test, *Q* values for evaluation were as follows: cimetidine *Q* = 80%, amlodipine *Q* = 75%, esomeprazole in acid not more than (NMT) 10% and in buffer not less than (NLT) *Q* (*Q* = 75%), glibenclamide *Q* = 70%, metformin *Q* = 70%, metformin extended release tablet (within 1, 3, and 10 hours NLT 20–40%, 45–65%, and 85% of label claim, respectively), and rabeprazole *Q* = 75%.

For sildenafil tablets, the assay was performed according to Moriyasu et al*.*^42^ For each sample, three or six tablets were analyzed. The compliance range in the quantity test was set as follows: sildenafil tablets contain NLT 90% and NMT 110% (average of 3–6 units), and no unit contains less than 75% or more than 125% of the labeled amount of sildenafil. The compliance range in the dissolution test was an average dissolution rate (for 3 or 6 units) equal to or greater than 75%, with no unit showing less than 50% dissolution, at a dissolution time of 15 minutes. The content uniformity test could not be conducted because of insufficient material.

Rabeprazole assay was performed according to the method described in the approval information document of rabeprazole (in Japanese; available from http://www.info.pmda.go.jp/go/interview/1/580591_2329028F2054_1_009_1F) prepared by the rabeprazole manufacturers of Japan for health practitioners and provided by the PMDA. For each sample, five tablets were analyzed. The compliance range in the quantity test was set as follows: rabeprazole tablets contain NLT 90% and NMT 110% (average of 5 units), and no unit contains less than 75% or more than 125% of the labeled amount of rabeprazole. Because of insufficient material, the dissolution test was performed on only one unit per sample, and the Q value was 75%.

### Price.

The prices of all samples were recorded in local currency (KHR riel). The price per unit was then converted to USD. As recommended by the WHO/HAI manual, the mean supplier prices from the Management Sciences for Health (MSH) 2011, 2012, and 2013 international medical products price guide were taken as international reference prices.^43,44^ Prices for the different strengths of medicine were calculated individually for each of the samples.

### Data analysis.

For comparison of two groups, the *t*-test or Fisher’s exact test was performed using IBM SPSS 19 (SPSS Inc., Chicago, IL). The criterion of significance was taken as *P* < 0.05.

## RESULTS

A total of 372 samples of seven different medicines stated to be from 64 manufacturers in 16 countries, comprising cimetidine (*n* = 86), sildenafil (*n* = 30), amlodipine (*n* = 79), esomeprazole (*n* = 54), rabeprazole (*n* = 11), glibenclamide (*n* = 52), and metformin (*n* = 60) were collected and tested in this study. Most of the samples (61.3%) were collected in Phnom Penh (urban area) and the others were collected in Battambang, Kandal, Kampong Speu, and Takeo (rural areas). All of the collected samples were dispensed to the mystery shoppers without prescription. Imported samples accounted for 334 (89.8%) of the total. Details of the collected samples are summarized in Table 1.

**Table 1:** Overview of samples collected and analyzed from different outlets in Cambodia during 2011–2013

Year	Generic name	Number of samples, *n*	Area		Outlet				Manufacturing source	
			Urban, *n* (%)	Rural, *n* (%)	Dépôt A, *n* (%)	Dépôt B, *n* (%)	Pharmacy, *n* (%)	Wholesaler, *n* (%)	Domestic, *n* (%)	Imported, *n* (%)
2011	Cimetidine	86	57 (66.3)	29 (33.7)	19 (22.1)	34 (39.5)	29 (33.7)	4 (4.7)	29 (33.7)	57 (66.3)
	Sildenafil	30	5 (16.7)	25 (83.3)	2 (6.7)	14 (46.6)	12 (40.0)	2 (6.7)	0 (0.0)	30 (100)
2012	Amlodipine	79	45 (57.0)	34 (43.0)	13 (16.4)	27 (34.2)	32 (40.5)	7 (8.9)	1 (1.3)	78 (98.7)
	Esomeprazole	54	38 (70.4)	16 (29.6)	4 (7.4)	13 (24.1)	28 (51.8)	9 (16.7)	0 (0.0)	54 (100)
	Rabeprazole	11	10 (90.9)	1 (9.1)	0	2 (18.2)	8 (72.7)	1 (9.1)	0 (0.0)	11 (100)
2013	Glibenclamide	52	33 (63.5)	19 (36.5)	14 (26.9)	11 (21.2)	25 (48.1)	2 (3.8)	0 (0.0)	52 (100)
	Metformin	60	40 (66.7)	20 (33.3)	14 (23.3)	10 (16.7)	31 (51.7)	5 (8.3)	8 (13.3)	52 (86.7)
Total		372 (100)	228 (61.3)	144 (38.7)	66 (17.7)	112 (30.1)	165 (44.4)	29 (7.8)	38 (10.2)	334 (89.8)

### Results of observation.

Samples were checked for 46 items according to the FIP tool for visual inspection of medicines.^36^ Eleven items needed to be verified by manufacturers or compared with authentic medicines, and a few items could not be checked because of the condition of collected samples, such as no boxes (sold in primary packaging). Among the major observed anomalies, one manufacturer had two different package designs (layout and/or printed colors) for the same brand of cimetidine. Another manufacturer had three different packaging designs for three samples with the same brand name. Plastic bottle and box packaging for two different samples of same brand from one manufacturer were observed, and the color of the tablets and the format of the attached document were different. The company name above the registration number was misspelled. There was a hole in one of the blisters of one sildenafil sample. This might have been due to mismanagement at the shop. Another sildenafil sample was found with a registration number printed directly on the box that was different from the number affixed with a seal. This might have been because the box was not changed at the shop when new products were brought in. Seven amlodipine samples, one esomeprazole sample, and one glibenclamide sample did not have a registration label on the box. Three amlodipine samples and two esomeprazole samples were found with no package insert, and for one metformin sample, the address provided on the package insert did not match that on the outer package. One esomeprazole sample was observed with no lot number, date of manufacture, or expiry date. Five metformin samples included a cracked tablet. Foreign flakes inside the blister, pink spots on the tablets, and nonuniformity of tablet shape were also observed in the case of metformin. Details of the visual observation and packaging analysis results are presented in Supplemental Table 2.

### Authenticity, legitimacy investigation, and registration verification.

Each manufacturer and each manufacturing country were sent a questionnaire and a request to verify the authenticity and legitimacy of the product and the manufacturer, respectively; however, the responses to our requests were unsatisfactory, as had previously been the case.^5,13,27^ Most manufacturers had a contact e-mail address, but in most cases, replies were not received even after a reminder e-mail to nonresponders. Among the 82 questionnaires sent to the manufacturers of 372 samples and 91 brands, responses were received for only 25 (30.5%) questionnaires, confirming the authenticity of 107 (28.8%) samples. The authenticity of the remaining samples (71.2%) could not be verified and remains unknown. Of 36 questionnaires sent to 16 manufacturing countries in total, only 14 (38.9%) responses were received from the MRAs confirming the legitimacy and manufacturing approval of 23 (28.1%) manufacturers to manufacture the medicines. Most of the samples (364; 97.8%) were properly registered with DDF, Cambodia, for marketing and distribution. Two manufacturers of glibenclamide and three manufacturers of metformin were confirmed not to have registration numbers and one manufacturer of sildenafil could not be verified. Interestingly, one manufacturer of glibenclamide confirmed their sample to be authentic, but the product was not registered in Cambodia. The registration status of one manufacturer of sildenafil was withdrawn by the DDF, Cambodia, although we collected several samples from this manufacturer. The results of authenticity investigation are presented in Table 2 and registration verification in Table 3 with details of the individual samples.

**Table 2:** Results of authenticity investigation of the collected samples

Year	INN	Number of samples, *n*	Manufacturers, *n*	Brands, *n*	Manufacturers replied, *n* (%)	Reply on samples, *n* (%)	Authentic, *n* (%)		
							Yes	No	Unknown
2011	Cimetidine	86	16	16	6 (37.5)	25 (29.1)	25 (29.1)	0	61 (70.9)
	Sildenafil	30	9	10	1 (11.1)	3 (10.0)	3 (10.0)	0	27 (90.0)
2012	Amlodipine	79	21	22	8 (38.1)	27 (34.2)	27 (34.2)	0	52 (65.8)
	Esomeprazole	54	16	22	3 (18.8)	13 (24.1)	13 (24.1)	0	41 (75.9)
	Rabeprazole	11	1	2	1 (100.0)	11 (100.0)	11 (100.0)	0	0 (0.0)
2013	Glibenclamide	52	4	4	1 (25.0)	2 (3.8%)	2 (3.8%)	0	50 (96.2)
	Metformin	60	15	15	5 (33.3)	26 (43.3)	26 (43.3)	0	34 (56.7)
Total		372 (100%)	82	91	25 (30.5)	107 (28.8)	107 (28.8)	0	265 (71.2)

INN = international nonproprietary names.

**Table 3:** Legitimacy investigation and registration verification by DDF, Cambodia

Year	Generic	Number of samples	Manufacturers, *n* (%)	Manufacturing country, n	MRA replied, *n* (%)	Reply on manufacturers, *n* (%)	Legitimacy, *n* (%)			Registration in Cambodia, *n* (%)	
							Yes	No	Unknown	Manufacturer	Sample
2011	Cimetidine	86	16	7	4 (57.1)	6 (37.5)	6 (37.5)	0	10 (62.5)	16 (100.0)	86 (100.0)
	Sildenafil	30	9	2	1 (50.0)	1 (11.1)	1 (11.1)	0	8 (88.9)	8 (88.9)	29 (96.7)
2012	Amlodipine	79	21	8	4 (50.0)	8 (38.1)	8 (38.1)	0	13 (61.9)	21 ((100.0)	79 (100.0)
	Esomeprazole	54	16	4	2 (50.0)	3 (18.8)	3 (18.8)	0	13 (81.2)	16 (100.0)	54 (100.0)
	Rabeprazole	11	1	1	1 (100.0)	1 (100.0)	11 (100.0)	0	0 (0.0)	1 (100.0)	11 (100.0)
2013	Glibenclamide	52	4	4	0 (0.0)	0 (0.0)	0 (0.0)	0	4 (100.0)	2 (50.0)	48 (92.3)
	Metformin	60	15	10	2 (20.0)	4 (26.7)	4 (26.7)	0	11 (73.3)	12 (80.0)	57 (95.0)
Total		372 (100%)	82	36	14 (38.9)	23 (28.1)	23 (28.1)	0	59 (71.9)	76 (92.3)	364 (97.8)

### Pharmaceutical analysis results.

Table 4 shows the results of chemical analysis of each of the 372 samples collected from Cambodia during 2011–2013. Overall, 76.6% (95% CI 72–81) of samples were found to be compliant and 23.4% (95% CI 19–28) of samples were noncompliant according to the predefined criteria. In the case of rabeprazole tablets collected in 2012, all the samples (100%) were found to be compliant, although only five tablets and one tablet were analyzed for quantity and dissolution, respectively, because of insufficient material. Among other medicines, 36.0% (95% CI 26–47) of the cimetidine samples in 2011 were noncompliant in some respect. Notably, more than half (53.7%) of the esomeprazole samples analyzed were noncompliant in 2012; 25.9% (95% CI 15–40) that gave values below 30% in the buffer stage of the dissolution test.

**Table 4:** Results of chemical analyses

Year	Generic	Number of samples, *n*	Quantity, *n* (%)		Content uniformity, *n* (%)		Dissolution, *n* (%)		All pass or any fail, *n* (%)	
			Compliant	Noncompliant	Compliant	Noncompliant	Compliant	Noncompliant	Compliant	Noncompliant
2011	Cimetidine	86	71 (82.6)	15 (17.4)	65 (75.6)	21 (24.6)	79 (91.9)	7 (8.1)	55 (64.0)	31 (36.0)
	Sildenafil	30	30 (100.0)	0 (0.0)	Not tested*	Not tested*	17 (56.7)	2 (6.7)†	28 (93.3)	2 (6.7)
2012	Amlodipine	79	78 (98.8)	1 (1.2)	73 (92.4)	6 (7.6)	77 (97.5)	2 (2.5)	72 (91.1)	7 (8.9)
	Esomeprazole	54	33 (61.1)	21 (38.9)	32 (59.3)	22 (40.7)	31 (57.4)	22 (40.7)‡	25 (46.3)	29 (53.7)
	Rabeprazole	11	11 (100.0)	0 (0.0)	Not tested*	Not tested*	11 (100)	0 (0.0)	11 (100.0)	0 (0.0)
2013	Glibenclamide	52	46 (88.5)	6 (11.5)	49 (94.2)	3 (5.8)	47 (90.1)	5 (8.9)	41 (78.8)	11 (21.2)
	Metformin	60	55 (91.7)	5 (8.3)	60 (100.0)	0 (0.0%)	58 (96.7)	2 (3.3)	53 (88.3)	7 (11.7)
Total		372 (100%)	324 (87.1)	48 (12.9)	320 (86.0)	52 (14.0)	320 (86)	40 (10.8)§	285 (76.6)	87 (23.4)

Results of identification test are not shown, as all the samples were identified as containing the respective API.

* Not tested because of the insufficient number of units.

† Dissolution test for 11 sildenafil samples was not performed because of the limited number of units.

‡ Dissolution test for one esomeprazole samples was not performed because of the limited number of units.

§ Dissolution test for a total of 12 (3.2%) samples was not performed because of the limited number of units.

**Figure 1.**
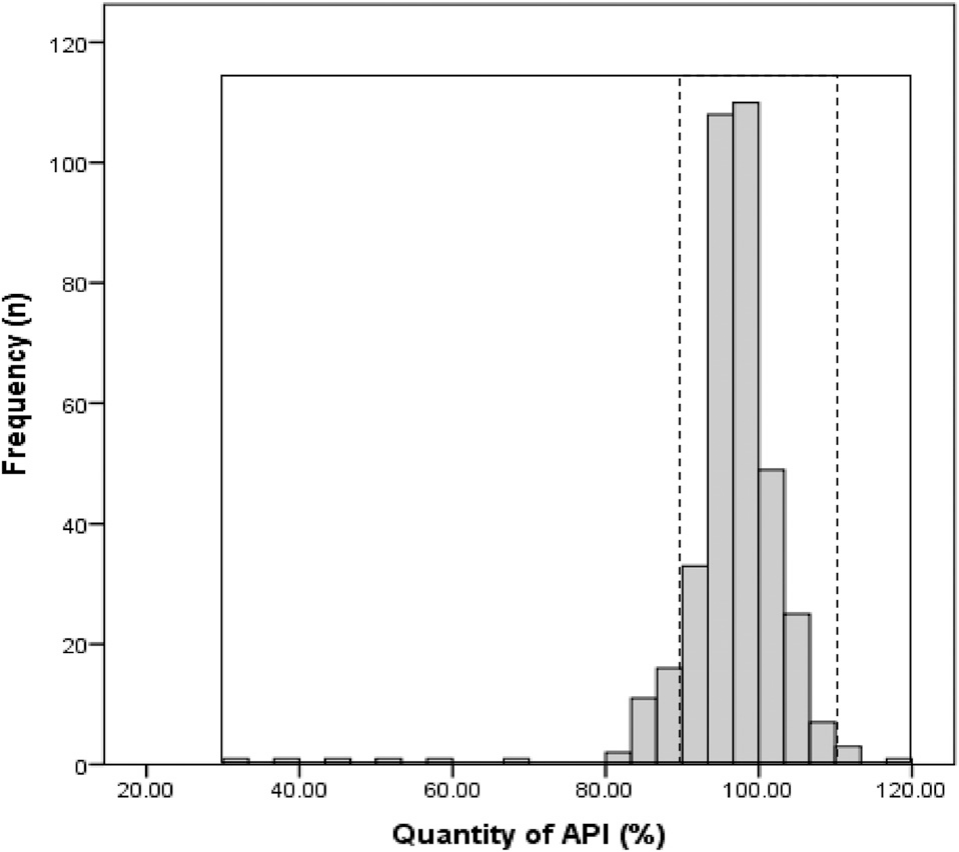
Frequency of the mean %API found in the samples (*N* = 372). Dashed lines represent 90–110% cutoff and solid lines, 30–120%.

**Figure 2.**
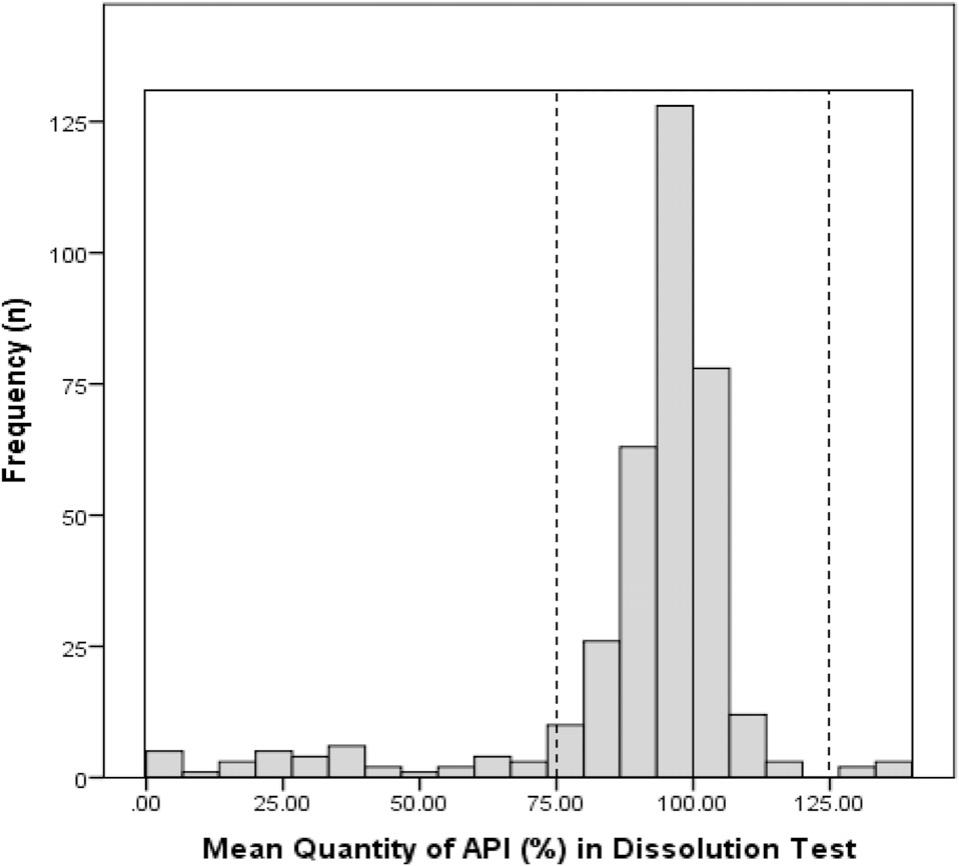
Frequency of the mean %API dissolved in the dissolution medium in the dissolution test (*N* = 360). Dashed lines represent 75–125% cutoff and solid lines, 0.90–140%.

Several samples showed extreme deviation from the acceptance criteria. For example, in the quantity test, the mean %API of one cimetidine and one amlodipine sample was 37% and 69% of the stated amount, respectively. Similarly, five esomeprazole samples contained less than 50% of the stated amount of API. In the dissolution test, one (1.2%, 95% CI 0.0–6.0) cimetidine, 20 (37.0%, 95% CI 24–51) esomeprazole, and three (5.8%, 95% CI 1.0–16) glibenclamide samples released less than 50% of the declared amount of the API. Figure 1 shows the frequency of the mean API in the quantity test of all samples from 2011 to 2013 (*n* = 372), whereas Figure 2 shows the frequency of the mean API dissolved in the medium in the dissolution test. Mean API of esomeprazole in the acid stage is not included in the chart, as none of the samples gave a value of more than 10%.

### Factors potentially influencing the outcome of the quality test.

#### Price and quality of medicines.

**Figure 3.**
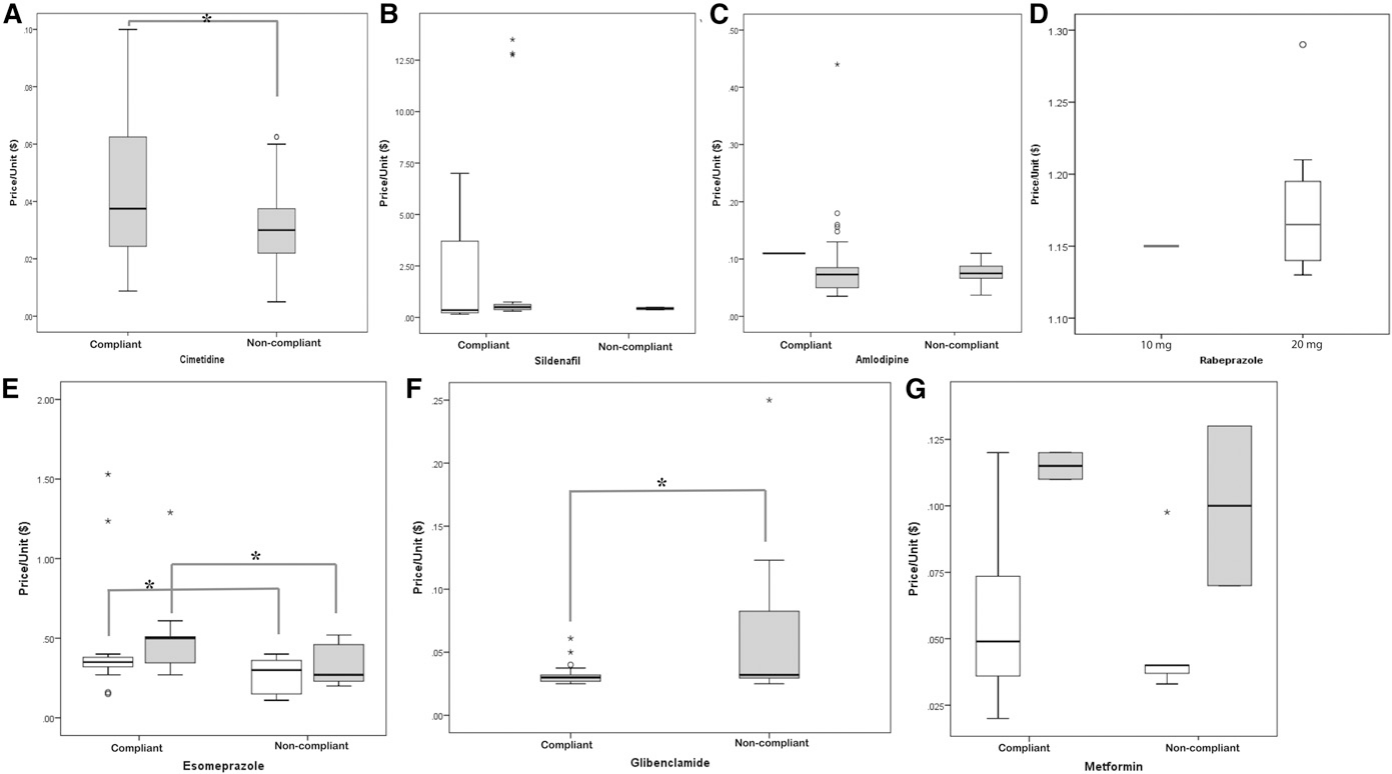
Price vs. quality of medicines. The panels compare the mean prices of different strengths of compliant and noncompliant samples; the significance of differences was evaluated using the *t*-test. (**A**) Cimetidine (only 400 mg of cimetidine samples were collected). (**B**) White bar represents 50 mg sildenafil samples and gray bar represents 100 mg samples. (**C**) White bar represents 10 mg amlodipine samples and gray bar represents 5 mg samples. (**D**) White bar represents 20 mg rabeprazole and gray bar represents 10 mg rabeprazole (**E**) White bar represents 20 mg esomeprazole samples and gray bar represents 40 mg samples. (**F**) Glibenclamide (only 5 mg glibenclamide samples were collected). (**G**) White bar represents 500 mg metformin samples and gray bar represents 850 mg samples. Rabeprazole data were not compared, as all rabeprazole samples were compliant.

The prices of samples were compared with those of MSH.^44^ The mean price of all the cimetidine samples seemed to be little higher than the reference price^44^ and the prices of the compliant cimetidine samples were higher than those of the noncompliant samples (*P* < 0.05, *t*-test, Supplemental Table 3). Similar situations were observed for sildenafil, amlodipine, esomeprazole, and metformin, where the mean prices of compliant samples were higher than those of the noncompliant samples (Figure 3, Supplemental Table 3). The price of the noncompliant 5 mg glibenclamide samples was higher than that of the compliant samples (*P* < 0.05, *t*-test). Several samples were outliers on the high side (Figure 3).

#### Storage conditions and other related factors.

In total, 82.0% samples were stored in the shops without air-conditioning. For 9.4% of the samples, mystery shoppers could not establish the presence or absence of air-conditioning (for details, see Supplemental File 7). However, we found no significant difference of storage temperature or humidity between compliant and noncompliant samples, as shown in Supplemental Figure 1.

The outcome of the quality test was not influenced by the location of sample collection, except in the case of amlodipine, where samples collected in rural areas showed a greater failure rate than samples collected from urban areas (*P* < 0.05, Supplemental Table 4). Relatively few samples were manufactured domestically, but among them, cimetidine samples showed a higher failure rate than imported samples (*P* < 0.05, Supplemental Table 5). One sample of amlodipine had been manufactured locally; it was noncompliant. Among seven domestic metformin samples, one was noncompliant. There appeared to be no difference in quality, depending on whether the samples were collected from a retailer or a wholesaler (Supplemental Table 6).

## DISCUSSION

This study focused on medicines for NCDs and the results have confirmed the circulation of poor-quality medicines in both urban and rural areas of Cambodia. Visual inspection revealed various issues, such as sample packaging variation, misspelling, missing registration label, cracked tablets, foreign particles, stains and nonuniform shape of tablets (Supplemental Table 2). These findings could be suggestive of falsification, particularly in the case of variation in packaging type for the same brand, misspelling of the manufacturer’s name, or the presence of foreign particles inside the blister. Even though no pass–fail decision was derived from the visual observation test, most of the samples showing anomalies in the test were found to be noncompliant in the pharmacopoeial analysis. Thus, failure to pass the visual observation test could be predictive of failure in the pharmacopoeial tests. Another important concern was the hot and humid environment in shops without air-conditioning, as these medicines should be stored below 30**°**C in a dry place, protected from light. Only 8.60% of samples were collected from shops equipped with air-conditioning and most of the remaining samples were simply stored in an unprotected environment. It seems likely that unsuitable storage conditions would have affected the quality of these medicines, even though we did not find that the presence or absence of air-conditioning was significantly related to the outcome of the quality tests.

No obviously falsified medicines were detected in this study. However, the response to the authenticity investigation (questionnaire survey of manufacturers and MRAs) was as low as 28.8% for all samples, and this issue seems to be a low priority for manufacturers and MRAs.^27,45^ For effective monitoring of falsified medicines, it is indispensable to get better cooperation from manufacturers and governments of manufacturing countries.

As regards quality, among the 372 samples collected from 2011 to 2013 in Cambodia, 23.4% (95% CI 19–28) samples were found to be noncompliant in at least one of the quantity, content uniformity, and dissolution tests. The most common failure was in the assay and content uniformity tests, except for enteric-coated esomeprazole capsules, where there was a high failure ratio in the buffer stage of the dissolution test (40.7%, 95% CI 28–55), including three samples showing below 5% release of esomeprazole. Overall, among the 54 collected samples of esomeprazole, 53.7% (95% CI 40–67) were noncompliant in some respect. The MoH, Cambodia, began to implement a mandatory requirement of dissolution testing for registration status in 2011 for some medicines, which may help to improve the situation. The result for esomeprazole is consistent with a previous report on omeprazole, in which it was suggested that some manufacturers might not yet have the technical capability to reliably produce enteric-coated preparations.^13^ For cimetidine samples in 2011, there was a high failure rate because of variation of content (24.6%, 95% CI 16–35) followed by the assay, where 17.4% (95% CI 10–27) of samples failed to meet the pharmacopoeial requirement, resulting in 36.0% (95% CI 26–47) unacceptability in total. These results suggest inadequate formulation or manufacturing processes of these medicines. Very large discrepancies were observed for some samples, including one esomeprazole sample with only 30% and one cimetidine sample with 37.0% of the claimed API. Several other samples contained less than 60% of the claimed API, although we could not establish whether these samples were falsified. There was also a great variation of the quantity of API within samples and between their units. It is often difficult to differentiate substandard and degraded medicines because of unavailability of samples, original preparations, or sufficient chemical information to distinguish them.^4^ Perhaps, we need clear definitions of how little API in a dosage unit should be regarded as substandard. Any sample showing deviation from this range should be evaluated as being falsified. It is suggested that in addition to the requirements set by the importing countries, exporting countries should also act to prevent the export of substandard medicines.

In most cases, the prices of the noncompliant samples were lower than those of the compliant samples. For example, the mean prices of noncompliant cimetidine, and the 20 and 40 mg esomeprazole samples were significantly lower than those of compliant samples. Similarly, the mean prices of noncompliant 100 mg sildenafil, 5 mg amlodipine, and the 500 and 850 mg metformin samples were lower than those of compliant samples. Apparently, medicines with low prices are more likely to be of poor quality. However, the opposite was the case for glibenclamide samples, where mean price of the noncompliant samples was significantly higher than that of compliant samples. So, it is not clear whether low prices of medicines necessarily imply poor quality of drugs.

### Limitations of the study.

It should be noted that the data are a few years old and, thus, may not fully reflect the current situation. Other limitations of this study include the restricted areas of sample collection, insufficient availability of some samples during the stratified random sampling in Phnom Penh, and the use of convenience sampling from selected drug outlets in rural areas. In addition, most of the manufacturers and MRAs failed to respond to the authenticity and legitimacy investigation; therefore, we could not establish whether most samples and manufacturers were authentic or not. Furthermore, dissolution test for 12 samples were not conducted in our quality evaluation because of an insufficient number of samples. But, although these limitations make it difficult to assess the actual extent of SFs in the entire Cambodian supply chain, our results establish that there is a substantial proportion of SFs among the investigated medicines.

## CONCLUSION

Our findings show that substandard medicines are circulating in the market in Cambodia, and there is an urgent need for routine monitoring to improve the quality of medicines for NCDs. Rabeprazole, sildenafil, amlodipine, and metformin were generally of satisfactory quality, but there was marked inter-unit variation among cimetidine samples and insufficient dissolution of the API in many esomeprazole samples. Strengthening the regulatory requirements for registration of the products to be imported into the country might be helpful, and the MRAs in the exporting countries could play a significant role in this respect. The storage conditions of these medicines generally did not meet the required standards. Better cooperation from manufacturers and MRAs is needed in the future to facilitate authenticity investigation.

## Supplemental file, tables, and figure

Supplemental materials
