# Supplementary material for: A Cross-Sectional Investigation of the Quality of Selected Medicines for Noncommunicable Diseases in Private Community Drug Outlets in Cambodia during 2011–2013
Source: Am J Trop Med Hyg. 2019 Sep 16;101(5):1018–26. doi: 10.4269/ajtmh.19-0247 (PMC6838583; doi:10.4269/ajtmh.19-0247)

Supplementary Figure 1: Storage versus Quality of Medicines. White represents the mean storage temperature of the samples during collection in °C and grey represents relative humidity in percent.

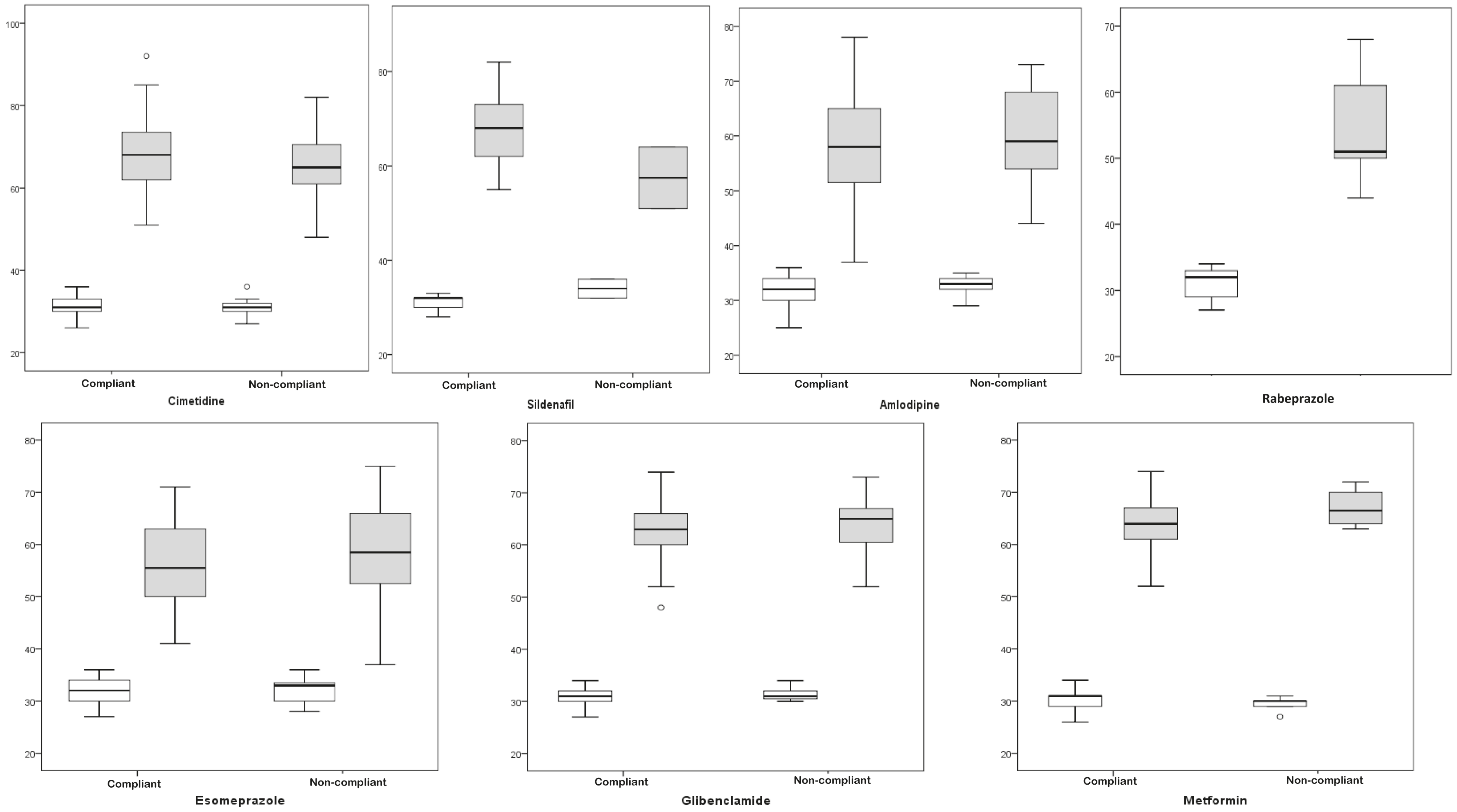

Supplement: Supplementary file 1 [file tpmd190247.SD1.pdf]
