# Supplementary material for: A Cross-Sectional Investigation of the Quality of Selected Medicines for Noncommunicable Diseases in Private Community Drug Outlets in Cambodia during 2011–2013
Source: Am J Trop Med Hyg. 2019 Sep 16;101(5):1018–26. doi: 10.4269/ajtmh.19-0247 (PMC6838583; doi:10.4269/ajtmh.19-0247)
Supplement: Supplementary file 2 [file tpmd190247.SD2.docx]

**S1 Table: Chromatography and dissolution conditions for chemical analysis of the collected samples**

| **Year** | **INN** | **Internal standard** | **Pharmacopoeia** | **Chromatographic conditions** | | | | | | **Dissolution conditions** | | | |
| --- | --- | --- | --- | --- | --- | --- | --- | --- | --- | --- | --- | --- | --- |
|  |  |  |  | **Mobile Phase (v/v)** | **Column** | **Flow rate** | **Wavelength** | **Oven temp.** | **Injection volume** | **Medium** | **Temp.** | **Apparatus** | **RPM-time** |
| 2011 | Cimetidine | Famotidine | USP 34 | Methanol: pH 5.6 phosphoric acid buffer = 1:4 | CLC-ODS 4.6 mm×25 cm column | 1.0 mL/min | 220 nm | 40℃ | 10 μL | 0.01 M HCl | 37 ℃ | Basket (apparatus 1) | 100 rpm-15 minutes |
|  | Sildenafil | Sulfadoxine | Moriyasu *et al* [40] | 0.05 mM phosphate buffer: acetonitrile = 73:27 | CLC-ODS 4.6 mm×15 cm column | 1.0 mL/min | 290 nm | 40℃ | 10 μL | 0.1 M HCl | 37 ℃ | Basket (apparatus 1) | 100 rpm-15 minutes |
| 2012 | Amlodipine | Isobutyl p-Hydroxybenzoate | USP 34 | Methanol, acetonitrile, and pH 3 Trimethylamine buffer = 7:3:10 | CLC-ODS 4.6 mm×15 cm column | 1.0 mL/min | 237 nm | 40℃ | 50 μL | 0.01 N hydrochloric acid | 37 ℃ | Paddle (apparatus 2) | 50rpm-30 minutes |
|  | Esomeprazole | Lansoprazole | USP 35 | Phosphate buffer pH 7.3:Acetonitrile: water = 50:35:15 | CLC-ODS 4.6 mm×15 cm column | 1.0 mL/min | 302 nm | 30℃ | 10 μL | Acid stage-0.1 N hydrochloric acid | 37 ℃ | Paddle (apparatus 2) | Acid stage, 100 rpm-120 minutes |
|  |  |  |  |  |  |  |  |  |  | Buffer stage-pH 6.8 phosphate buffer |  |  | Buffer stage, 100 rpm-30 minutes |
|  |  |  | BP 2012 | Phosphate buffer pH 6.8: Acetonitrile= 6:4 |  | 0.5 mL/min |  |  |  | Acid stage- pH 4.5 phosphate buffer solution |  |  | Acid stage, 100 rpm-45 minutes |
|  |  |  |  |  |  |  |  |  |  | Buffer stage-pH 7.6 phosphate buffer |  |  | Buffer stage, 100 rpm-45 minutes |
|  | Rabeprazole | 1-Amino-2-methylnaphthalene | PMDA, JP | Phosphate buffer pH 7: Methanol = 2:3 | Mightysil RP-18 GP 4.6 mm×15 cm column | 1.0 mL/min | 290 nm | 30℃ | 10 μL | Tris hydroxymethyl amino methane pH 9.0 | 37 ℃ | Paddle (apparatus 2) | 100-60 minutes |
| 2013 | Glibenclamide | Progesterone | USP 35 | Water: Acetonitrile= 11:9 and 4.0 mL of  phosphoric acid per L of solution | CLC-ODS 4.6 mm×15 cm column | 2.0 mL/min | 215 nm | 25℃ | 50 μL | Borate buffer, pH 9.5 | 37 ℃ | Paddle (apparatus 2) | 75 rpm-45 minutes |
|  |  | Butylparaben | BP 2012 | Phosphate buffer pH 3:Acetonitrile = 1:1 |  | 1.0 mL/min | 225 nm | 25℃ | 20 μL | Phosphate buffer pH 7.8 | 37 ℃ |  | 100 rpm-45 minutes |
|  | Metformin | - | USP 35 | Phosphate buffer pH 3:Acetonitrile = 1:1 | CLC-ODS 4.6 mm×25 cm column | 0.9 mL/min | 233 nm | 30℃ | 10 μL | Phosphate buffer pH 6.8 | 37 ℃ | Basket (apparatus 1) | 100 rpm-45 minutes |
|  |  | - | BP 2012 | Phosphate buffer pH 3:Acetonitrile = 1:1 | CLC-ODS 4.6 mm×25 cm column | 0.9 mL/min | 232 nm | 30℃ | 10 μL | Phosphate buffer pH 6.8 | 37 ℃ | Basket (apparatus 1) | 100 rpm-45 minutes |
|  | Metformin-Extended Release | - | USP 35 | Heptanesulfonate and sodium chloride buffer pH 3.85: Acetonitrile=9:1 | CLC-ODS 4.6 mm×25 cm column | 0.9 mL/min | 218 nm | 30℃ | 10 μL | Phosphate buffer pH 6.8 | 37 ℃ | Paddle (apparatus 2) | 100 rpm-60, 180, and 600 minutes |
