# Supplementary material for: A Cross-Sectional Investigation of the Quality of Selected Medicines for Noncommunicable Diseases in Private Community Drug Outlets in Cambodia during 2011–2013
Source: Am J Trop Med Hyg. 2019 Sep 16;101(5):1018–26. doi: 10.4269/ajtmh.19-0247 (PMC6838583; doi:10.4269/ajtmh.19-0247)
Supplement: Supplementary file 3 [file tpmd190247.SD3.docx]

**S2 Table : Visual observation and packaging analysis**

| **Generic** | **Number of Samples, n** | **Packaging Variation (Color design, color density, package type etc.), n** | **No Insert, n** | **Missing or Differences in Registration Label and Number, n** | **Information Variation in Package and Insert, n** | **Misspelling** | **No Lot No., MFD or EXD, n** | **Color Variation, Defective Tablet/Capsule, Defective Blister, n** |
| --- | --- | --- | --- | --- | --- | --- | --- | --- |
| **Cimetidine** | 86 | 10 | 0 | 0 | 0 | 1 | 0 | 4 |
| **Sildenafil** | 30 | 0 | 0 | 1 | 0 | 0 | 0 | 1 |
| **Amlodipine** | 79 | 0 | 3 | 7 | 0 | 0 | 1 | 0 |
| **Esomeprazole** | 54 | 0 | 2 | 1 | 0 | 0 | 1 | 0 |
| **Rabeprazole** | 11 | 0 | 0 | 0 | 0 | 0 | 0 | 0 |
| **Glibenclamide** | 52 | 0 | 0 | 1 | 0 | 0 | 0 | 0 |
| **Metformin** | 60 | 0 | 0 | 0 | 1 | 0 | 0 | 8 |
| **Total** | 372 | 10 (2.7%) | 5 (1.4%) | 10 (2.7%) | 1 (0.27%) | 1 (0.27%) | 2 (0.54%) | 13 (3.5%) |
