# Supplementary material for: A Cross-Sectional Investigation of the Quality of Selected Medicines for Noncommunicable Diseases in Private Community Drug Outlets in Cambodia during 2011–2013
Source: Am J Trop Med Hyg. 2019 Sep 16;101(5):1018–26. doi: 10.4269/ajtmh.19-0247 (PMC6838583; doi:10.4269/ajtmh.19-0247)
Supplement: Supplementary file 4 [file tpmd190247.SD4.docx]

**S3 Table: Price versus quality of medicines**

| **Year** | **Generic** | **Strength** | **Number of samples, n** | **Compliant** | **Price/Unit ($)**  **Mean±SD** | **Non-compliant, n** | **Price/Unit ($)**  **Mean±SD** | **p value (t-test)** |
| --- | --- | --- | --- | --- | --- | --- | --- | --- |
| 2011 | Cimetidine | 400 mg | 86 | 55 | 0.042± 0.022 | 31 | 0.032±0.014 | p < 0.05 |
|  | Sildenafil | 50 mg | 4 | 4 | 1.96±2.91 | 0 | 0 | n.t.^a^ |
|  |  | 100 mg | 26 | 24 | 2.047±4.15 | 2 | 0.425±0.05 | 0.592 |
| 2012 | Amlodipine | 10 mg | 2 | 2 | 0.11±0.0 | 0 | 0 | n.t.^a^ |
|  |  | 5 mg | 77 | 69 | 0.078±0.053 | 7 | 0.076± 0.021 | 0.898 |
|  | Esomeprazole | 20 mg | 28 | 13 | 0.477± 0.398 | 15 | 0.257± 0.11 | p < .05 |
|  |  | 40 mg | 26 | 12 | 0.506±0.257 | 14 | 0.333±0.12 | p < .05 |
|  | Rabeprazole | 10 mg | 1 | 1 | 1.15 | 0 | 0 | n.t.^a^ |
|  |  | 20 mg | 10 | 10 | 1.172±0.046 | 0 | 0 | n.t.^a^ |
| 2013 | Glibenclamide | 5 mg | 52 | 41 | 0.03±0.01 | 11 | 0.07±0.07 | p < .001 |
|  | Metformin | 500 mg | 56 | 51 | 0.06±0.02 | 5 | 0.05±0.03 | 0.800 |
|  |  | 850 mg | 4 | 2 | 0.12±0.01 | 2 | 0.10±0.04 | 0.564 |

a. not tested
