# Supplementary material for: A Cross-Sectional Investigation of the Quality of Selected Medicines for Noncommunicable Diseases in Private Community Drug Outlets in Cambodia during 2011–2013
Source: Am J Trop Med Hyg. 2019 Sep 16;101(5):1018–26. doi: 10.4269/ajtmh.19-0247 (PMC6838583; doi:10.4269/ajtmh.19-0247)
Supplement: Supplementary file 5 [file tpmd190247.SD5.docx]

**S4 Table: Area versus Quality of Medicines**

| **Year** | **Generic** | **Number of samples, n** | **Urban** | | **Rural** | | **p value** |
| --- | --- | --- | --- | --- | --- | --- | --- |
|  |  |  | **Compliant** | **Non-compliant** | **Compliant** | **Non-compliant** |  |
| 2011 | Cimetidine^a^ | 86 | 37 | 16 | 18 | 11 | 0.624 |
|  | Sildenafil | 30 | 2 | 0 | 15 | 2 | n.t.^b^ |
| 2012 | Amlodipine | 78 | 44 | 1 | 27 | 6 | p < 0.05  p < 0.05 |
|  | Esomeprazole | 53^c^ | 18 | 19 | 6 | 10 | 0.454 |
|  | Rabeprazole | 11 | n.t.^b^ | | | | |
| 2013 | Glibenclamide | 52 | 28 | 5 | 13 | 6 | 0.181 |
|  | Metformin | 60 | 36 | 4 | 17 | 3 | 0.676 |
| Total | | 372 (100%) |  |  |  |  |  |

a: 4 cimetidine samples were collected from a wholesaler; b: not tested; c: Dissolution test for one esomeprazole sample was not done because of the limited number of units
