# Supplementary material for: A Cross-Sectional Investigation of the Quality of Selected Medicines for Noncommunicable Diseases in Private Community Drug Outlets in Cambodia during 2011–2013
Source: Am J Trop Med Hyg. 2019 Sep 16;101(5):1018–26. doi: 10.4269/ajtmh.19-0247 (PMC6838583; doi:10.4269/ajtmh.19-0247)
Supplement: Supplementary file 6 [file tpmd190247.SD6.docx]

**S5 Table: Origin versus Quality of Medicines**

| **Year** | **Generic** | **Number of samples, n** | **Domestic** | | **Imported** | | **p value** |
| --- | --- | --- | --- | --- | --- | --- | --- |
|  |  |  | **Compliant** | **Non-compliant** | **Compliant** | **Non-compliant** |  |
| 2011 | Cimetidine^a^ | 86 | 12 | 17 | 43 | 14 | p < 0.01  p < 0.05 |
|  | Sildenafil | 30 | a | | | | |
| 2012 | Amlodipine | 79 | 1 | 0 | 70 | 7 | n.t.^b^ |
|  | Esomeprazole | 54 | c | | | | |
|  | Rabeprazole | 11 | d | | | | |
| 2013 | Glibenclamide | 52 | e | | | | |
|  | Metformin | 60 | 7 | 1 | 46 | 6 | 1.000 |
| Total | | 372 (100%) |  |  |  |  |  |

a: All sildenafil samples were of foreign origin; b not tested; c: All esomeprazole samples were of foreign origin; d: All esomeprazole samples were of foreign origin; e: All glibenclamide samples were of foreign origin
