# Supplementary material for: A Cross-Sectional Investigation of the Quality of Selected Medicines for Noncommunicable Diseases in Private Community Drug Outlets in Cambodia during 2011–2013
Source: Am J Trop Med Hyg. 2019 Sep 16;101(5):1018–26. doi: 10.4269/ajtmh.19-0247 (PMC6838583; doi:10.4269/ajtmh.19-0247)
Supplement: Supplementary file 7 [file tpmd190247.SD7.docx]

**S6 Table 6: Retailer versus Wholesaler**

| **Year** | **Generic** | **Number of samples, n** | **Retailer** | | **Wholesaler** | | **p value** |
| --- | --- | --- | --- | --- | --- | --- | --- |
|  |  |  | **Compliant** | **Non-compliant** | **Compliant** | **Non-compliant** |  |
| 2011 | Cimetidine^a^ | 86 | 54 | 28 | 1 | 3 | 0.131 |
|  | Sildenafil | 30 | 24 | 2 | 4 | 0 | n.t.^a^ |
| 2012 | Amlodipine^d^ | 79 | 64 | 7 | 7 | 0 | n.t.^a^ |
|  | Esomeprazole | 54 | 21 | 23 | 3 | 6 | 0.340 |
|  | Rabeprazole | 11 | n.t.^b^ | | | | |
| 2013 | Glibenclamide | 52 | 40 | 10 | 1 | 1 | 0.382 |
|  | Metformin | 60 | 49 | 6 | 4 | 1 | 0.475 |
| Total | | 372 (100%) |  |  |  |  |  |

a: not tested; b: not tested (all rabeprazole samples were of compliant)
